# IRF2BP2-deficient microglia block the anxiolytic effect of enhanced postnatal care

**DOI:** 10.1038/s41598-017-10349-3

**Published:** 2017-08-29

**Authors:** Aswin Hari, Shelly A. Cruz, Zhaohong Qin, Pascal Couture, Ragnar O. Vilmundarson, Hua Huang, Alexandre F. R. Stewart, Hsiao-Huei Chen

**Affiliations:** 1 Ottawa Hospital Research Institute, Ottawa, Canada; 2 University of Ottawa, Brain and Mind Institute, Ottawa, Canada; 3 University of Ottawa Heart Institute, Ottawa, Canada; 4 Biochemistry, Microbiology, and Immunology, University of Ottawa, Ottawa, Canada; 5 Cellular and Molecular Medicine, University of Ottawa, Ottawa, Canada; 6 Medicine, University of Ottawa, Ottawa, Canada; 7 Canadian Partnership for Stroke Recovery, Ottawa, Canada; 8 University of Ottawa, Center for Infection, Immunity and Inflammation (CI3), Ottawa, Canada

## Abstract

Enhanced postnatal care (EPC) increases resilience to adversity in adulthood. Since microglia participate in shaping neural circuits, we asked how ablation of an inflammation-suppressing factor IRF2BP2 (Interferon Regulatory Factor 2 Binding Protein 2) in microglia would affect the responses to EPC. Mice lacking IRF2BP2 in microglia (KO) and littermate controls (WT) were subjected to EPC during the first 3 weeks after birth. EPC reduced anxiety in WT but not KO mice. This was associated with reduced inflammatory cytokine expression in the hypothalamus. Whole genome RNAseq profiling of the hypothalamus identified 101 genes whose expression was altered by EPC: 95 in WT, 11 in KO, with 5 in common that changed in opposite directions. Proteoglycan 4 (Prg4), prostaglandin D2 synthase (Ptgds) and extracellular matrix protease inhibitor Itih2 were suppressed by EPC in WT but elevated in KO mice. On the other hand, the glutamate transporter VGLUT1 (Slc17a7) was increased by EPC in WT but not KO mice. Prostaglandin D2 (PGD2) is known to enhance microglial inflammation and promote Gfap expression. ELISA confirmed reduced PGD2 in the hypothalamus of WT mice after EPC, associated with reduced Gfap expression. Our study suggests that the anxiety-reducing effect of EPC operates by suppressing microglial inflammation, likely by reducing neuronal prostaglandin D2 production.

## Introduction

Mammalian infants are born in an immature state; neural connections are continually formed and actively remodelled in the first 2–3 weeks after birth^1^. Maternal care during this critical period of brain development has long-term consequences and natural differences in maternal care are reflected in the behaviours of their adult progeny. For example, increased maternal care is associated with reduced anxiety and improved social behaviour in the offspring^2, 3^. Experimental manipulations that enhance postnatal care (EPC), such as handling and tactile stimulation of pups and/or short-term maternal separation to increase maternal licking/care after reunion with their pups, reduces anxious behaviours in adulthood^4–8^. The mechanisms whereby EPC modulates neural circuits to produce enduring changes in emotional behaviours are not fully understood.

Emerging evidence points to an important role for microglia in shaping neural networks during the early postnatal period^1, 9^. Microglia are specialized brain-resident macrophages, important innate immune cells that guard the health of the brain^10^. Microglia prune away unwanted synapses in an activity-dependent process, whereby neurons tag unwanted synapses with complement proteins so that they can be eliminated by microglia^1, 11^. In addition, chemokines, cytokines and growth factors produced from microglia can directly signal to neurons and astrocytes to regulate their functions^12–14^. Reciprocally, neurons signal back to microglia to modulate microglia function^15^. Furthermore, by modulating the extracellular matrix surrounding neurons^16^ microglia also indirectly influence synaptic connectivity and plasticity^17^.

Like macrophages, microglia can become polarized to an M1 state and produce pro-inflammatory cytokines including IL1β, IL6 and TNFα, or to an M2 state and produce anti-inflammatory cytokines IL4 and IL10^10^. Our studies have identified interferon regulatory factor 2 binding protein 2 (IRF2BP2) as a key regulator of macrophage and microglial polarization^18, 19^. IRF2BP2-deficient microglia have heightened expression of M1 inflammatory cytokines in response to challenge with bacterial lipopolysaccharides (LPS) and impaired activation of the anti-inflammatory M2 marker genes in response to IL4 stimulation^19^. The contribution of M1 or M2 microglia to the shaping of neural networks during development is an important question. Here, we sought to address whether altered microglia polarization could affect the outcome of EPC on adult emotionality. Our study indicates that IRF2BP2-deficient microglia block the EPC-mediated suppression of anxiety and points to prostaglandin D2 involvement in this process.

## Results

### EPC reduced anxiety in WT but not KO mice

**Figure 1:**
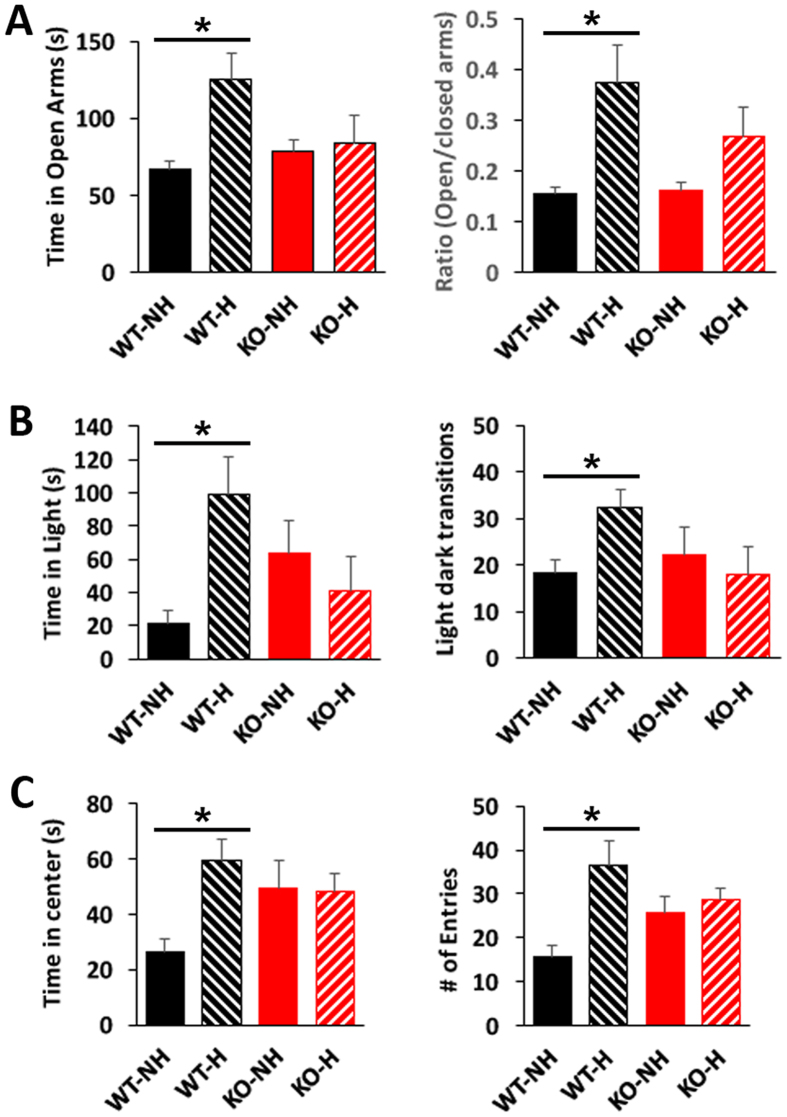
Enhanced postnatal care (EPC) reduces anxiety in WT but not KO mice. Anxiety behaviours measured by (**A**) elevated plus maze, (**B**) light/dark preference, and (**C**) open field tests reveal reduced anxiety in WT mice after EPC (WT-H) compared to non-handled mice (WT-NH). N = 9 mice per group, *p < 0.05 by post-hoc comparison with Bonferroni correction after two-factor ANOVA.

After 3 weeks of EPC by postnatal handling, a battery of behaviour tests to evaluate anxiety was carried out in male mice, starting at the age of 5 weeks. There was no significant difference in anxiety between non-handled WT and KO mice. However, WT male mice exhibited reduced anxiety after postnatal handling: spending more time in the open arms of the EPM (Fig. 1A), more time in the lighted area of the light-dark preference test (Fig. 1B) and more time in the center in the open field test (Fig. 1C). On the other hand, KO mice did not show a difference in anxiety phenotypes after handling. By two-factor ANOVA, the effect of handling was significant (F:6.159, degree of freedom (df):1, p = 0.019), as was the interaction between genotype and handling (F:4.190, df:1, p = 0.049) in the EPM test. In the light-dark preference test, a significant interaction between genotype and handling was observed for the time spent in the light (F: 9.093, df:1, p = 0.006). In the open field test, for both time in the center and the number of entries to the center, a significant effect of handling (F:4.643, df:1, p = 0.041; F:7.981, df:1, p = 0.009) and the interaction between genotype and handling (F:5.290, df:1, p = 0.03; F:5.611, df:1, p = 0.026) were also observed. Although there is a trend toward less anxiety in WT after EPC (WT-H) compared to KO after EPC (KO-H), the difference did not reach statistical significance after correcting for multiple testing.

### EPC had no effect on fear conditioning

**Figure 2:**
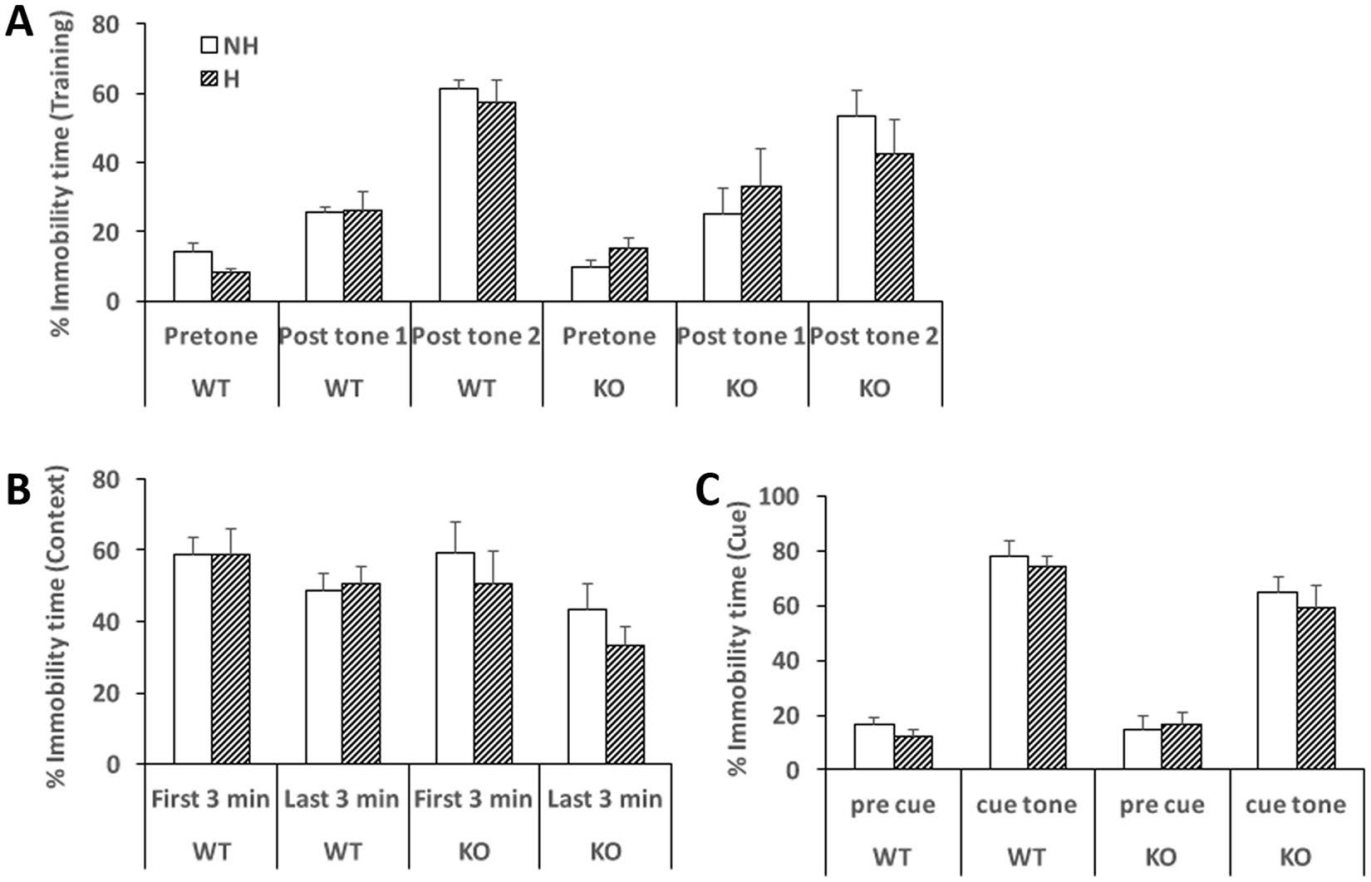
No effect of EPC on fear conditioning. Both WT and KO mice acquire a conditioned fear in response to tone/shock pairing on training day 1 (**A**), as revealed by context-induced freezing on day 2 (**B**) and increased response to tone cue on day 3 (**C**). No difference was observed with EPC (H, handled) compared to non-handled (NH) controls. N = 9 mice per group.

To determine whether neonatal handling affects fear conditioning, we conducted a fear conditioning protocol over three days at 7 weeks of age, including a training day with a tone-shock, a context day without tone-shock and a cue day when mice were placed in a novel environment before exposure to the tone from the training day. Both WT and KO mice acquired fear conditioning, as seen in the increased immobility responses to tone in Fig. 2C (~80%) compared to Fig. 2A (~20 and 60% for post-tone 1 and 2, respectively). However, EPC had no effect on the response of either WT or KO mice.

### EPC reduced depression caused by social defeat in both WT and KO mice

**Figure 3:**
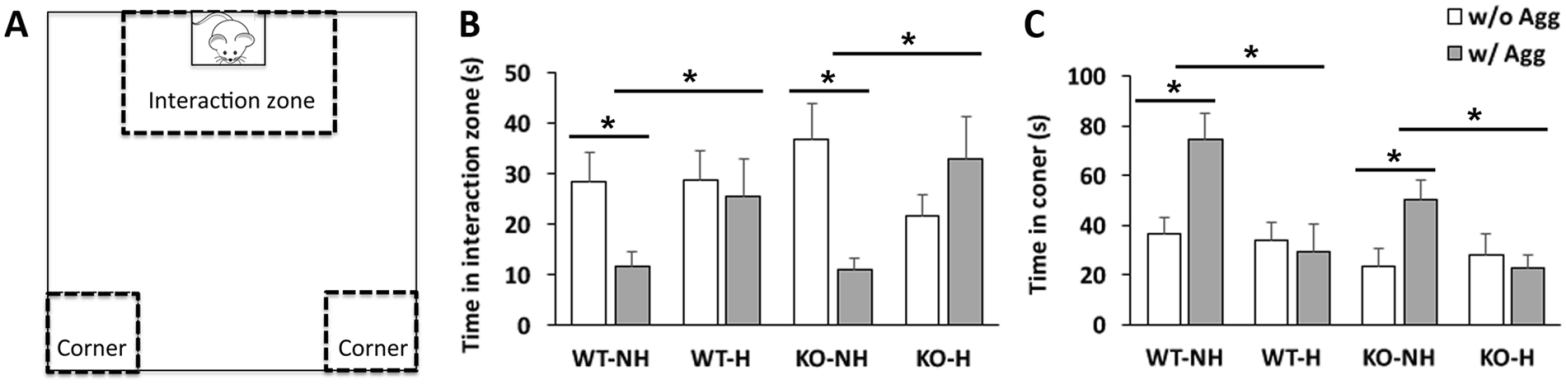
EPC improves resilience to social defeat in both WT and KO mice. Arena diagram (**A**) shows area of interaction and corners in the social defeat test. After 10 days repeated social defeat, non-handled WT and KO mice spent less time in the interaction zone (**B**) and more time spent in the corners (**C**) when an aggressive mouse is present (w/Agg). EPC increased interaction with an aggressor and reduced time hiding in the corners in both WT and KO mice. N = 9 mice per group, *p < 0.05 by post-hoc comparison with Bonferroni correction after two-factor ANOVA.

At 9 weeks, male mice were subjected to 10 days of repeated social defeat by exposure to an aggressive CD1 male mouse. A standard social interaction test (Fig. 3A) was carried out the following day to measure resiliency and depression after social defeat. Both WT and KO unhandled mice display susceptibility to social defeat and depression evidenced by reduced time spent in the interaction zone in the presence of an aggressor (Fig. 3B, compare white to grey bars) and increased time spent hiding in the corners (Fig. 3C, compare white to grey bars). EPC increased resilience to social defeat in both WT and KO mice (F:19.60, df:1, p = 0.0001), as seen by increased time interacting with the aggressor (Fig. 3B, compare grey bar) and less time spent in the corners (Fig. 3C, compare grey bars). However, EPC-induced resilience was not different in WT or KO mice.

### EPC suppressed inflammatory genes in the hypothalamus of WT but not KO mice

**Figure 4:**
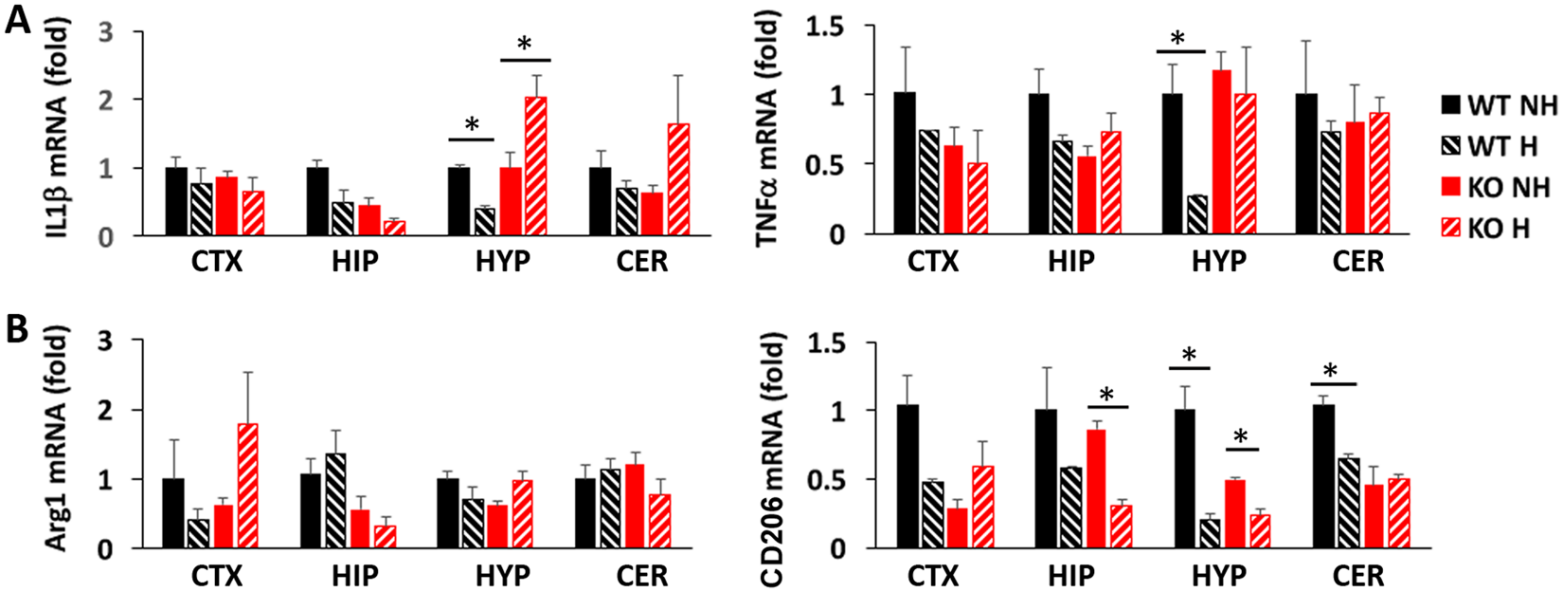
EPC reduces inflammatory cytokine expression in the hypothalamus of WT but not KO mice. mRNA expression of inflammatory (**A**) and anti-inflammatory (**B**) markers was determined by RT-qPCR for different brain regions (CTX, cortex; HIP, hippocampus; HYP, hypothalamus; CER, cerebellum). All values were normalized to actin and expressed as a fold of non-handled WT mice. N = 3 mice per group. *p < 0.05 by unpaired t-test.

Since anxiety has been tied to brain inflammation^20, 21^, we postulated that EPC might reduce anxiety by suppressing inflammatory genes in microglia. We analysed various brain regions including the cerebral cortex (CTX), the hippocampus (HIP), the hypothalamus (HYP) and the cerebellum (CER) at 5 weeks by qPCR for the expression of inflammatory cytokines IL1β and TNFα (M1 inflammatory markers) and the anti-inflammatory M2 markers Arg1 and CD206 (Fig. 4). A significant reduction in inflammatory markers was observed in WT hypothalamus, but not other brain regions, after EPC. In contrast, KO hypothalamus showed an increase in ILβ and no change in TNFα in response to EPC. Intriguingly, expression of the anti-inflammatory marker CD206 was also reduced in both WT and KO hypothalamus after EPC.

### Transcriptomic profiling of genes altered by EPC in the hypothalamus

**Figure 5:**
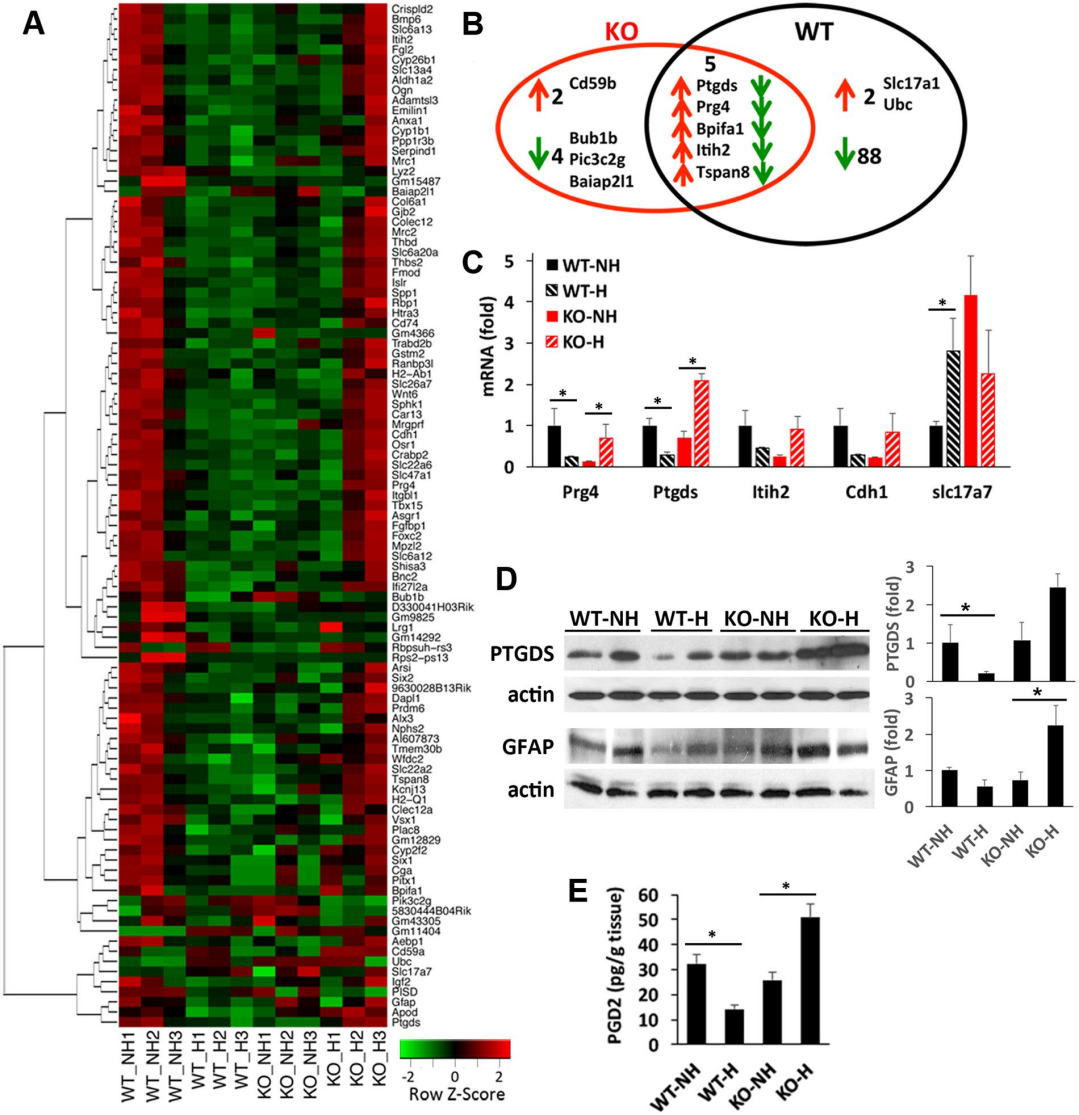
Transcriptomic profiling of hypothalamus identifies genes differentially regulated by EPC in WT and KO mice. (**A**) Heat map of 101 differentially expressed transcripts identified by RNAseq. N = 3 mice per group. (**B**) Venn diagram showing the number of differentially expressed genes in KO and WT hypothalamus after EPC. Arrows indicate direction of change after EPC. (**C**) RT-qPCR confirmed altered expression of 5 genes of interest in the hypothalamus. (**D**) Representative immunoblots of hypothalamic protein extracts shows reduced expression of Ptgds and Gfap in WT mice after EPC (WT-H) compared to controls (WT-NH). The opposite change was observed in KO. Blots were quantified and normalized to actin, expressed as fold change relative to NH-WT. N = 4 mice per group. *p < 0.05 by unpaired t-test. (**E**) ELISA assay of PGD2 levels in hypothalamus. N = 4 mice per group. *p < 0.05 by unpaired t-test. Two-way ANOVA revealed a significant interaction between genotype and treatment on PGD2 levels (F = 32.3, df:1, p = 0.00046).

Informed by our qPCR analysis of inflammatory and anti-inflammatory genes in different brain regions, unbiased genome-wide transcriptomic RNAseq profiling was used to compare gene expression in WT and KO hypothalamus in handled and non-handled mice at 5 weeks. A heatmap of the 101 differentially expressed transcripts is shown in Fig. 5A (also see Supplementary Table S1). A Venn diagram illustrates genes altered by EPC: 11 genes in KO and the 95 genes in WT, with the 5 overlapping genes all being oppositely regulated in WT and KO mice (Fig. 5B). qPCR was used to confirm differential expression of 5 transcripts in a separate cohort of mice (Fig. 5C). Pathway analysis revealed altered gene expression by EPC in 4 functional categories (Table 1): Proteoglycans and associated proteins of the extracellular matrix, membrane transporters, retinoic acid signalling and Wnt signalling.

**Table 1:** Pathway analysis of genes altered by EPC.

Rank	Gene	WT (H/NH)	KO (H/NH)	WT (H/NH)	KO (H/NH)
		log_FC	log_FC	p value*	p value*
Proteoglycans and associated proteins of the extracellular matrix					
2	Prg4	−3.716	4.123	4.80E-07	0.015
4	Itih2	−2.155	1.831	0.0025	0.015
10	Cdh1	−3.417	2.892	8.20E-06	0.13
18	Fmod	−2.94	2.102	0.00022	0.34
21	Ogn	−2.968	2.175	0.00026	0.31
29	Serpind1	−1.967	1.821	0.00091	0.13
40	Thbd	−1.529	1.044	0.0022	0.82
42	Islr	−1.522	1.17	0.0022	1
55	Adamtsl3	−1.441	0.883	0.0056	1
67	Lrg1	−1.912	2.755	0.012	0.31
78	Thbs2	−0.8	0.295	0.02	1
Membrane transporters					
11	Slc17a7	1.493	−0.887	1.80E-05	1
14	Slc47a1	−2.767	2.241	0.00011	1
15	Slc22a6	−3.243	2.36	0.00021	0.55
19	Slc26a7	−2.441	1.524	0.00025	1
20	Kcnj13	−3.946	1.636	0.00026	1
22	Slc13a4	−3.013	2.292	4.00E-04	0.13
45	Slc6a12	−3.243	2.764	0.0027	0.47
50	Slc6a13	−2.237	1.858	0.0038	0.12
71	Slc6a20a	−1.624	1.206	0.017	1
83	Slc22a2	−3	2.428	0.029	0.47
Retinoic acid signalling					
12	Osr1	−3.284	2.999	3.00E-05	0.13
27	Aldh1a2	−2.505	2.288	7.00E-04	0.13
32	Apod	−0.531	0.387	0.0015	1
41	Cyp26b1	−1.631	0.714	0.0022	1
56	Crabp2	−2.319	2.587	0.0064	0.13
64	Rbp1	−1.025	0.906	0.01	1
Wnt signalling					
10	Cdh1	−3.417	2.892	8.20E-06	0.13
24	Wnt6	−2.471	1.873	0.00044	0.55
53	Itgbl1	−2.073	1.833	0.0048	0.49
63	Trabd2b	−1.453	0.973	0.01	1
68	Shisa3	−1.401	0.407	0.013	1
82	Emilin1	−1.335	0.636	0.025	1
85	Plac8	−3.255	0.508	0.029	1

Log_FC, log_2_ fold change. *EdgeR adjusted p value. N = 3 male mice/group.

Ptgds, the gene coding for prostaglandin D2 synthase, is one of the most highly expressed transcripts that showed opposite change by EPC in WT and KO mice. Immunoblot analysis of hypothalamic extracts from a different cohort of mice confirmed lower Ptgds protein levels in WT and increased Ptgds protein levels in KO that were handled compared to littermates that were not (Fig. 5D). The hypothalamic prostaglandin D2 levels, measured by ELISA (Fig. 5E), correlated with changes seen in Ptgds mRNA (Fig. 5C) and protein levels (Fig. 5D). Prostaglandin D2 has been reported to increase expression of Gfap in astrocytes^12^. Indeed, we observed reduced expression of Gfap by RNAseq in WT hypothalamus after EPC (Fig. 5A, Supplementary Table S1), and this was confirmed by immunoblot (Fig. 5D, Supplemental Fig. S1).

## Discussion

Our study showed that EPC reduced anxiety and increased resilience to social defeat, but had no effect on fear conditioning in mice. Importantly, we found that ablation of IRF2BP2 in microglia hinders the anxiolytic effect of EPC, but not the EPC-induced resilience to social defeat. These observations indicate that different neural circuits are involved in these emotional responses, where anxiety is affected by IRF2BP2 in microglia while resilience to social stress is not. It should be noted that our findings do not exclude a contribution of microglia in shaping EPC-induced resilience to social stress. Microglia in different brain regions are known to differ in their gene expression profiles^22^ and loss of IRF2BP2 may only affect a subset of microglia.

Since EPC reduced the basal expression of inflammatory cytokines in the hypothalamus of WT but not KO mice, and this was associated with reduced anxiety in WT but not KO mice, we postulate that basal inflammatory tone in the hypothalamus sets the level of anxiety in mice. It is noteworthy that adult mice with M1 polarized inflammatory microglia in the hypothalamus display anxiety^20, 21^. An important question is whether IRF2BP2 expression in microglia of the hypothalamus is regulated (elevated) by EPC. Addressing this question would require isolation of microglia pooled from the hypothalamus of large numbers of handled versus non-handled sex-matched mice of known genotype born at the same time. This is a technical challenge and limitation of our study design. It should be noted that microglia numbers in the hypothalamus were not different by genotype or by EPC treatment (Supplemental Fig. S2). Nonetheless, failure to suppress inflammation might explain why mice with IRF2BP2-deficient microglia fail to respond to the anxiolytic effect of EPC.

The hypothalamus is a key region that controls the stress response and anxiety^23^. The hypothalamus showed the most robust change of inflammatory cytokines by EPC in our qPCR screening of different brain regions. Neonatal handling is known to improve the response of the Hypothalamus-Pituitary-Adrenal (HPA) axis to stress in adulthood, with a more rapid return of stress-induced hormones to baseline, without changing basal levels of stress responsive hormones between handled and non-handled animals^24^. In line with these observations, our RNAseq analysis did not reveal a significant change following EPC in the expression of CRH (corticotropin-releasing hormone) or AVP (arginine vasopressin), two key hormones of the hypothalamic stress response, or in the levels of their receptors. On the other hand, RNAseq identified many hypothalamic genes that are differentially altered by EPC between WT and KO mice that could account for their different anxiety response in adulthood.

Prostaglandin D2 synthase (Ptgds) is highly expressed and showed marked opposite altered expression in response to EPC in WT and KO mice. Ptgds converts prostaglandin H2 to prostaglandin D2 and is predominantly expressed in the leptomeninges, choroid plexus and oligodendrocytes of the adult brain^25^. In line with Ptgds mRNA and protein levels, hypothalamic PGD2 levels were lower with EPC in WT, but elevated in KO mice. PGD2 has proinflammatory effects; it worsens LPS-induced inflammatory cytokine production in macrophages^26^, activates microglia and exacerbates neuronal damage^27^. PGD2 promotes Gfap expression, an index of inflammatory astrogliosis^12, 28^. Indeed, we found reduced Gfap expression in WT hypothalamus after EPC concomitant with reduced Ptgds and PGD2 levels. Intriguingly, mice exposed to long-term environmental enrichment have reduced anxiety and lowered Ptgds expression^29^. Thus, Ptgds is likely one of the mechanisms whereby EPC reduces inflammation in the hypothalamus to lower anxiety.

The extracellular matrix is a degradable dynamic microenvironment that not only regulates the growth of dendritic spines but also modulates synaptic plasticity^17^. Several components of the extracellular matrix were differentially altered by EPC between WT and KO mice, including Prg4, Itih2, Cdh1, Fmod, Adamtsl3, Thbd, and Thbs2. Prg4 is a chondroitin sulphate proteoglycan and Itih2 (inter-alpha-trypsin inhibitor heavy chain 2) is a serine protease inhibitor involved in extracellular matrix stabilization. Prg4 and Itih2 both bind to hyaluronan^30, 31^ and the presence of hyaluronic acid at the peri-synaptic area influences use-dependent synaptic plasticity through regulation of dendritic calcium channels^32^. A close relative of ITIH2, ITIH3, has been tied to schizophrenia^33^. Cdh1 (E-cadherin) is well known for its role in synapse formation^34^. Both fibromodulin (Fmod) and thrombomodulin (Thbd) regulate complement activation^35, 36^ and could affect microglia-mediated synaptic pruning^1, 11^. Mice with global knockout of Fmod have improved cognitive performance^37^. Thrombospondin 2 (Thbs2), another protein that regulates extracellular matrix assembly^38^, has been associated with general anxiety disorder^39^. ADAMTSL3, a metalloprotease with thrombospondin-like motif implicated in degradation of chondroitin sulphate proteoglycans and remodelling of the extracellular matrix, has also been tied to schizophrenia^40^. Whether these changes affect the anxiety circuit will require further investigation.

Retinoic acid signalling was another major signalling pathway showing altered expression in response to EPC. Elevated retinoic acid (RA) augments inflammatory cytokine effects^41^ and has been implicated in anxiety^42^ and depression^43^. Although Cyp26b1, the main enzyme that degrades RA in the hypothalamus was reduced, an overall reduction in the RA signaling pathway was observed in WT mice after EPC. Aldh1a2, a key enzyme that synthesizes RA, was reduced by EPC, as were Crabp2, Rbp1, Osr1 and ApoD. Crabp2, the Cellular Retinoic Acid Binding Protein 2, promotes transfer of RA to the nucleus^44^. The cytosolic retinol binding protein 1 (Rbp1) binds vitamin A (retinol) and mediates retinol oxidation to RA^45^. The transcription factor Osr1 and the apolipoprotein ApoD, known downstream targets of RA^46, 47^, were both reduced by EPC in WT mice, consistent with reduced RA signalling.

Both canonical and non-canonical Wnt signalling are important for synaptic plasticity and CNS development^48^. Overall, EPC appears to suppress non-canonical Wnt signalling in WT mice (with reduced levels of Wnt6^49^) and increase canonical Wnt signalling (with reduced expression of known inhibitors of canonical Wnt signalling: Cdh1^50^, Itgbl1^51^, Shisa3^52^, Trabd2b^53^, Emilin1^54^, and Plac8^55^). Wnt6 has been tied to schizophrenia and mood disorders^56^. The failure of KO mice to down-regulate these key inhibitors of canonical Wnt signalling in response to EPC argues that IRF2BP2 in microglia is required for this effect.

Many membrane transporters also showed differential expression in response to EPC. Slc17a7, the gene coding the vesicular glutamate transporter (aka, vGluT1), was markedly increased by EPC in WT but not KO hypothalamus. Importantly, vGluT1 is induced by antidepressant drugs^57^ whereas reduced vGluT1 is tied to anxiety and depression^58, 59^. Thus, increased vGluT1 expression likely mediates part of the anxiety-reducing effect of EPC. Other members of the Slc (solute carrier) family of transporters showed reduced expression with EPC, including Slc26a7, Slc47a1, Slc22a6, Slc13a4, Slc6a12, Slc6a13, Slc6a20a, and Slc22a2. Although the significance of these changes on neuronal excitability remains to be determined, it is noteworthy that Slc6a12 and Slc6a13 code for GABA transporters (GAT2, GAT3), and variants at Slc6a13 are tied to anxiety disorder^60^.

In summary, the present study strongly suggests that EPC induces an anxiolytic effect by suppressing inflammation in the hypothalamus. Microglia lacking IRF2BP2 have a pro-inflammatory phenotype^19^ that likely interferes with the inflammation-suppressing effect of EPC. To our knowledge, ours is the first study to demonstrate the requirement of anti-inflammatory microglia in the hypothalamus for enhanced postnatal care to induce an anxiolytic effect lasting into adulthood.

## Methods

### Mice

All mice were bred into the C57BL6 background for over 10 generations. Mice with a floxed allele of IRF2BP2 were bred with LysMCre mice that express Cre-recombinase in the myeloid lineage to generate mice lacking IRF2BP2 in macrophage and microglia, as described previously^18, 19^. The animal care and use committee of the University of Ottawa approved all procedures carried out in mice. All methods were performed in accordance with the relevant guidelines and regulations.

### Neonatal handling to enhance postnatal care (EPC)

Neonatal handling was done daily from post-natal day 3 to 21, between 13:00 and 16:00 hours. Mice were divided into two groups: 1) non-handled mice that were not disturbed except for regular cage changes and 2) mice that underwent EPC. For EPC, pups were removed from the nest, held gently by the experimenter and stroked with the index finger on the dorsal surface for 15 min. The mice were then placed in separate bedding on a heated pad in the same room as the dams for 1 hour every day, so that the dams could hear their vocalizations. Both handled and non-handled mice were weaned on post-natal day 21 and genotyped. Only male mice were selected for further experiments and group-housed with littermates before behaviour tests.

### Behaviour tests of emotionality

The elevated plus maze, the light/dark preference and the open field tests were carried out in the University of Ottawa animal behaviour core to measure anxiety, as we described in detail previously^61^.

#### Fear conditioning

The fear-conditioning regimen included training, context and cue protocols. On the first day (training), mice were placed in the fear conditioning apparatus for a total of 6 minutes. After the first two minutes, a tone was played for 30 sec ending with a 2 sec shock (0.45 mA). One minute following the shock, the same tone was played again for 30 sec ending with a 2 sec foot shock. For the remaining two minutes there was no tone or shock. The freezing behaviour of the animal was recorded throughout the 6 minutes. On the second day, mice were returned to the training cage to measure context-induced fear and freezing, without tones or shocks. On the third day, mice were placed in a new context where the fear-conditioning apparatus was modified with a new smell and changed arena walls. Mice immobility was monitored 3 minutes before and 3 minutes during the tone cue. Freezing behaviour is measured using Noldus video tracking software (Ethovision).

#### Repeated Social defeat

CD-1 male mice (Charles River Laboratories) were screened and selected for aggressive behaviour over 5 days. A single aggressor mouse was based in one half of a rat cage with a divided and a unique intruder tester C57BL/6 mouse was introduced into the side with the aggressor for 1–5 minutes a day to allow social defeat. The process was continued for 10 days with every tester mouse seeing a different aggressor every day. On day 11, the tester mice were placed in an open field apparatus and tested for social interaction in the presence or absence of a novel CD1 male placed in a small enclosure in the field. Susceptibility to social defeat by encountering the aggressor was measured by video monitoring of time spent exploring the enclosure in the presence and absence of the novel CD1 aggressor mouse and time spent in the corner zones, analysed by the Noldus software.

### RNA extraction and qPCR

Total RNA from various tissues of the brain was extracted using the Qiagen RNeasy Mini Kit (74104). RNA purity, integrity and concentration were determined using a nanodrop and an Agilent Bioanalyzer. Reverse transcription reactions were carried out using the 5X All-in-one RT kit (ABM) after a DNase step to remove genomic DNA. qPCR was conducted on Rotorgene and Corbett equipment using Evagreen qPCR master mix (ABM). The results were normalized to actin. qPCR primers used are: TNFα (F) 5′-CCACCACGCTCTTCTGTCTAC-3′, (R) 5′-AGGGTCTGGGCCATAGAACT-3′; IL1β (F) 5′-CAGGCTCCGAGATGAACAA-3′, (R) 5′-CCCAAGGCCACAGGTATTT-3′;Arg1 (F) 5′-TCACCTGAGCTTTGATGTCG-3′, (R) 5′-CTGAAAGGAGCCCTGTCTTG-3′; CD206 (F) 5′-CAAGGAAGGTTGGCATTTGT-3′, (R) 5′-CCTTTCAGTCCTTTGCAAGC. Prg4 (F) 5′-CCTTTTTACAGCAAGGGCGT-3′, (R) 5′-CATCTCCCTGCACAGCTTGA-3′; Ptgds (F) 5′-GAGTAAACGCAGGTGAGAGAAG-3′, (R) 5′-TCTTGAGAGTGACAGAGCAAAG-3′; Itih2 (F) 5′-AAGAGGGCAGAGAATGGAAAG-3′, (R) 5′-GATGGTCTCGGTGCTGATTT-3′, Cdh1 (F) 5′-CTGCTGCTCCTACTGTTTCTAC-3′, (R) 5′-TCTTCTTCTCCACCTCCTTCT-3′, slc17a7 (F) 5′-GTGGCTGCCCAAAGCTATTA-3′, (R) 5′-GGAACCACCCAGGAGAATAAAG-3′; actin: 5′-GCTTCTTTGCAGCTCCTTCG-3′, 5′-CCTTCTGACCCATTCCCACC-3′.

### Immunoblot analysis and ELISA

Protein from frozen hypothalamic wedges was extracted in RIPA buffer, size fractionated by SDS-polyacrylamide gel electrophoresis and analyzed by immunoblot as described previously^61^ using antibody to Ptgds (SAB2108049), Gfap (SAB2107063), and normalized to beta-actin (A2668) (all antibodies purchased from Sigma). Iba1 antibody is purchased from Wako (019–19741). Hypothalamic tissue prostaglandin D2 levels were measured by ELISA, according to the manufacturer’s protocol (Cayman).

### RNAseq

Total RNA from hypothalami of 5 week-old WT and KO male mice with or without EPC (n = 3 per group) was extracted using the Qiagen RNeasy Mini Kit (74104). RNA purity, integrity and concentration were determined on an Agilent Bioanalyzer. RNAseq library preparation, Illumina (HiSeq. 2500) sequencing (paired end, 2 × 150 cycle run) and bioinformatics analysis were all carried out at the McGill University and Genome Quebec Innovation Centre (MUGQIC). We applied multiple test correction using the Benjamini & Hochberg method to the p values computed by edgeR using the R function “p.adjust”. This function returns the false discovery rate (FDR) for every gene (that we label as “adjusted p values”). The FDR cutoff, i.e. the adjusted p values of edgeR, for our RNAseq data is 0.05. Heat maps were generated using the heatmap.2 function of the gplots R package (http://CRAN.R-project.org/package=gplots). Transcripts were ranked first according to whether they were differentially regulated in the handled versus non-handled mice in opposite directions for WT and KO mice both at an adjusted edgeR p < 0.05. Next, genes were ranked according to their adjusted edgeR p < 0.05 for handled WT littermate mice compared to non-handled control mice and corresponding results in KO mice generally showed no significant change in either the same or an opposite direction. Lastly, we identified genes significantly altered by EPC only in KO mice (see Supplementary Table S1). Pathways enriched by EPC were identified using the DAVID pathway analysis online software (https://david-d.ncifcrf.gov/).

### Statistical analysis

For behaviour tests, two-factor ANOVA was used to examine the effects of genotype, handling and their interaction on measured variables. Post-hoc comparisons of means were carried out by paired t-test with Bonferroni correction, and corrected p < 0.05 was considered significant. For RNAseq analysis, an adjusted edgeR p < 0.05 was considered significant. For qPCR and immunoblot analysis, for between-group comparisons of fold changes, values were normalized by log transformation and a two-tailed Student’s t test was applied. Differences in means were considered significant at p < 0.05.

## Electronic supplementary material

Supplementary Table S1

Supplementary figures
