# Supplementary material for: IRF2BP2-deficient microglia block the anxiolytic effect of enhanced postnatal care
Source: Sci Rep. 2017 Aug 29;7:9836. doi: 10.1038/s41598-017-10349-3 (PMC5575313; doi:10.1038/s41598-017-10349-3)
Supplement: Supplementary file 2 — Supplementary figures [file 41598_2017_10349_MOESM2_ESM.doc]

**IRF2BP2-deficient microglia block the anxiolytic effect of enhanced postnatal care**

***Running title:* Microglia mediate the anxiolytic effect of enhanced postnatal care**

Aswin Hari^1,3*^, Shelly A. Cruz^1*^, Zhaohong Qin^1^, Pascal Couture^1^, Ragnar O. Vilmundarson^3^, Hua Huang^1,3^, Alexandre F. R. Stewart^3^, Hsiao-Huei Chen^1,2^

**Supplementary Figure S1 Full-size immunoblots shown in Fig. 5D.** Blue outlines indicate the regions cropped.

**Supplementary Figure S2 Immunofluorecent staining showed no significant difference in the numbers of microglia in the hypothalamus between WT and KO mice or after EPC.** Microglia (immune-positive for Iba1, in red) at the hypothalamic region were counted from 6 serial cryostat sections (20 μm thick) sampled every 10 sections, 4 different fields per coronal section were counted. Nuclei were counterstained by DAPI (in blue). N= 3 mice per group. Scale bar, 50 μm.

**
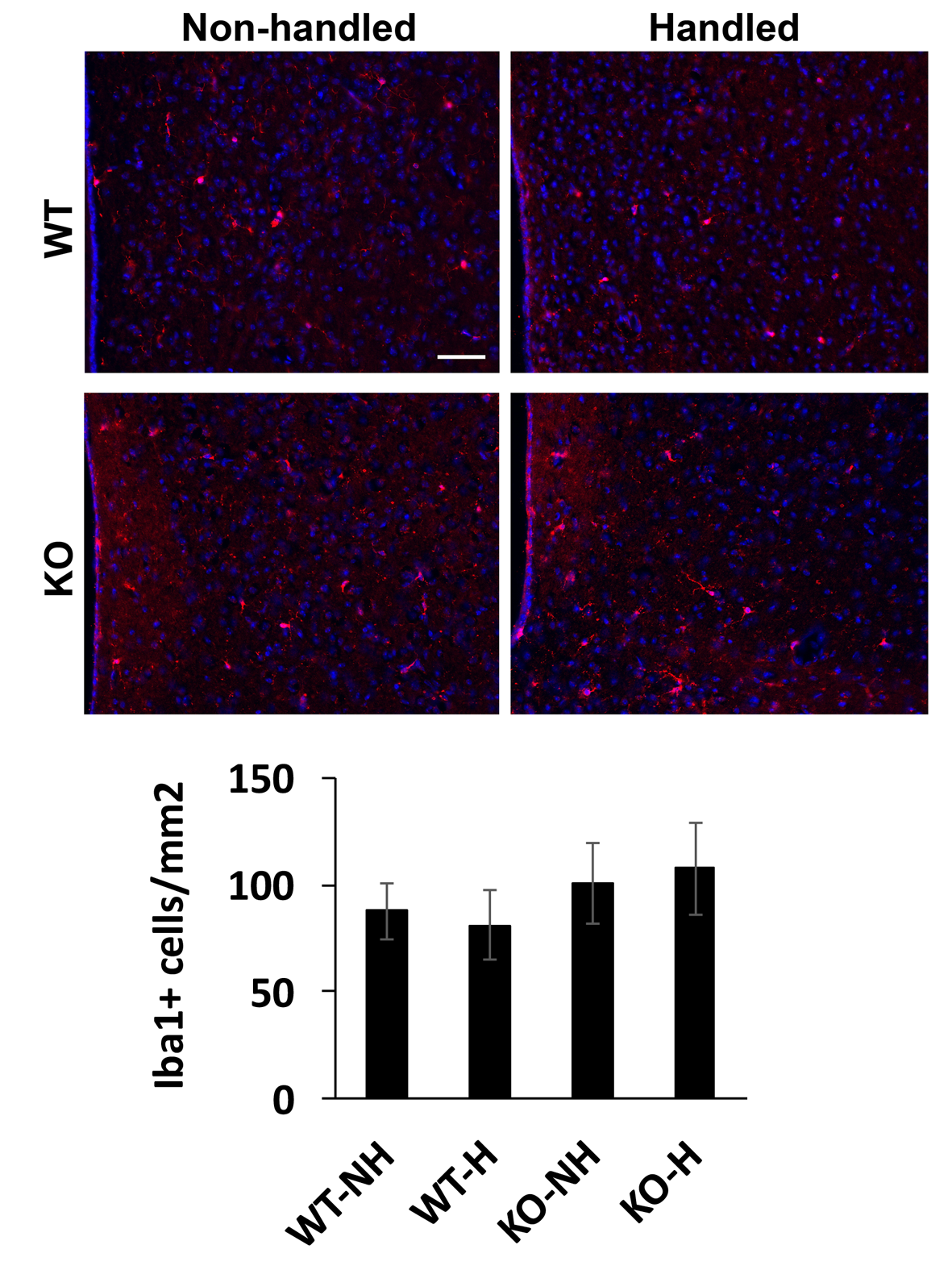
**
